# Long-term behavioral effects of prenatal stress in the Fmr1-knock-out mouse model for fragile X syndrome

**DOI:** 10.3389/fncel.2022.917183

**Published:** 2022-10-27

**Authors:** Valeria Petroni, Enejda Subashi, Marika Premoli, Maurizio Memo, Valerie Lemaire, Susanna Pietropaolo

**Affiliations:** ^1^Univ. Bordeaux, CNRS, INCIA, UMR 5287, F-33000 Bordeaux, France; ^2^Department of Molecular and Translational Medicine, University of Brescia, Brescia, Italy

**Keywords:** gene-environment interactions, prenatal stress, neurodevelopmental disorders, aging, social behaviors, ultrasonic communication, autism

## Abstract

Fragile X syndrome (FXS) is a major neurodevelopmental disorder and the most common monogenic cause of autism spectrum disorder (ASD). FXS is caused by a mutation in the X-linked FMR1 gene leading to the absence of the FMRP protein, inducing several behavioral deficits, including motor, emotional, cognitive, and social abnormalities. Beside its clear genetic origins, FXS can be modulated by environmental factors, e.g., stress exposure: indeed the behavioral phenotype of FXS, as well as of ASD patients can be exacerbated by the repeated experience of stressful events, especially early in life. Here we investigated the long-term effects of prenatal exposure to unpredictable chronic stress on the behavioral phenotype of the Fmr1-knock-out (KO) mouse model for FXS and ASD. Mice were tested for FXS- and ASD-relevant behaviors first at adulthood (3 months) and then at aging (18 months), in order to assess the persistence and the potential time-related progression of the stress effects. Stress induced the selective emergence of behavioral deficits in Fmr1-KO mice that were evident in spatial memory only at aging. Stress also exerted several age-specific behavioral effects in mice of both genotypes: at adulthood it enhanced anxiety levels and reduced social interaction, while at aging it enhanced locomotor activity and reduced the complexity of ultrasonic calls. Our findings underline the relevance of gene-environment interactions in mouse models of neurodevelopmental syndromes and highlight the long-term behavioral impact of prenatal stress in laboratory mice.

## Introduction

Fragile X syndrome (FXS) is a neurodevelopmental disorder due to a mutation in the X-linked FMR1 human gene leading to the absence of the FMRP protein (Pieretti et al., 1991), i.e., a key modulator of synaptic and neuronal functionality (Greenough et al., 2001). FXS is characterized by several behavioral abnormalities, including hyperactivity, anxiety, cognitive deficits (Hagerman and Hagerman, 2002), as well as social alterations, together with additional autistic symptoms (Bailey et al., 1998; Hagerman, 2006): FXS represents also the most common monogenic cause of autism spectrum disorder (ASD), so that preclinical models of FXS are often employed also to study ASD. This is the case of the Fmr1-KO mouse line, i.e., the most widely used animal model of FXS that recapitulates the lack of FMRP as well as most of the behavioral alterations observed in FX patients (The Dutch-Belgian Fragile X Consortium, 1994), including autistic-like behaviors (Bernardet and Crusio, 2006; Pietropaolo et al., 2011; Gauducheau et al., 2017). Although some behavioral alterations have been described in Fmr1-KO mice during development and adolescence (Bilousova et al., 2009; Spencer et al., 2011; Gaudissard et al., 2017; Kat et al., 2022), the most robust FXS-relevant behavioral phenotypes are mostly evident in mutant mice starting at early adulthood, i.e., at 3 months, that is, when most of the existing studies with the Fmr1-KO model have been performed (reviewed in Pietropaolo and Subashi, 2014). Little is instead known about the behavioral characteristics of Fmr1 mutants during aging; the persistence/stability of FXS-like symptoms is not an issue of obvious definition, since fluctuations in certain behavioral alterations (e.g., autistic symptoms) have been described in FXS patients with aging, while changes in cholinergic (Scremin et al., 2015) and endocannabinoid (Martin et al., 2017) functionality have been observed in aging Fmr1-KO mice. Furthermore, the brain expression of FMRP is known to be reduced in wild-type mice during aging, i.e., around 14 months of age, in parallel with their synaptic and behavioral decline (Singh et al., 2007; Prasad and Singh, 2008).

Despite their well-known genetic etiology, the behavioral symptoms of FXS can be markedly modulated by environmental factors, both in their severity and progression. While the exposure to environmental stimulation is able to attenuate/delay the appearance of behavioral alterations both in FXS patients and Fmr1-KO mice (Dawson et al., 2002; Oddi et al., 2015), the opposite effects have been described following stressful experiences. The chronic exposure to aversive and stressful events, especially during early life phases, is indeed known to exacerbate the behavioral symptoms of FXS patients (Hessl et al., 2001; Dyer-Friedman et al., 2002) and to anticipate the appearance of certain behavioral deficits in Fmr1-KO mice (Petroni et al., 2022). More in general, a large body of human research suggests that the offspring of mothers who experienced high levels of stress during pregnancy are more likely to have problems in their neurobehavioral development (reviewed in Van den Bergh et al., 2005; Rice et al., 2007). Prospective studies have for instance shown that prenatal maternal stress is able to increase in the offspring the risk for childhood behavioral and emotional problems, language delay, cognitive deficits, and several neurodevelopmental disorders. More specifically, the experience during pregnancy of family discord (Ward, 1990), stressful life events [recalled retrospectively; (Beversdorf et al., 2005)], hurricanes and tropical storms (Kinney et al., 2008a,b) have all been shown to be associated with elevated risk for ASD in the resulting offspring, as well as of ADHD (Beversdorf et al., 2005, 2019). Furthermore, it has been suggested that children with ASD exposed to prenatal stress may in general suffer from more severe behavioral symptoms than those with no history of prenatal stress (Varcin et al., 2017).

Based on this clinical and epidemiological data, it is evident that prenatal chronic stress represents a powerful environmental manipulation which is able to induce a strong early aversive experience. It is therefore likely that this aversive environmental factor may interact with the genetic risk of developing ASD and other neurodevelopmental pathologies, e.g., with the FMR1 mutation. It is thus surprising that the behavioral effects of stress have not been extensively investigated in the Fmr1-KO mouse model for ASD and FXS. The available studies have focused so far only on the short-term behavioral effects of chronic stress in Fmr1 mutants, either following prenatal (Petroni et al., 2022) or post-natal stress exposure (Qin et al., 2011; Lemaire-Mayo et al., 2017), reporting overall reduced adaptive responses to stress in KO mice. Furthermore, prenatal stress induced the appearance of certain behavioral deficits in juvenile Fmr1-KO males that were otherwise absent at this young age. Hence, stress could be a valuable tool to enhance the face validity of the Fmr1-KO mouse model for neurodevelopmental disorders, an issue that has been recently questioned (Kat et al., 2022), together with its predictive validity (Berry-Kravis et al., 2016). It is therefore important to assess whether these behavioral effects of prenatal stress were stable at the long-term, i.e., whether they could be detected also at adulthood and at aging in Fmr1 mutants.

Prenatal stress exposure is able to exert long-lasting behavioral alterations in wild-type rodents, i.e., inducing the emergence of cognitive, emotional, explorative, and social deficits at adulthood (reviewed in Weinstock, 2008; Sandi and Haller, 2015). Gestational stress has also shown in the rat and mouse offspring to accelerate the neurobehavioral decline associated with aging, particularly concerning cognitive abilities and emotional reactivity (Vallee et al., 1999; Grigoryan et al., 2019). In rodents, similarly to humans, aging appears in fact as a “fragile” period when the damage induced by early insults may gain further relevance (Koehl et al., 2001) and enhance its own ability to exert detrimental effects on the behavioral homeostasis of an individual (Koenig et al., 2011). This fragility may be particularly marked in the case of a concomitant genetic mutation, as in the case of FXS, thus maximizing the impact of gene-environment interactions on the pathological behavioral phenotype, as hypothesized for several neurodevelopmental and neurological disorders (Grossman et al., 2003). Hence, extending the behavioral evaluation of prenatal stress effects into the aging phase may allow to detect effects that were absent or marginal at adulthood and that may be “unmasked” by aging processes. In this context, it should be underlined that the long-term behavioral effects of prenatal stress in rodents may be mediated also by early post-natal factors, such as reduced maternal care of stressed dams and/or by raised corticosterone levels in their milk (Muir et al., 1985). In contrast to primates, a considerable amount of neural development occurs in the rat and mouse brain after birth, making it more sensitive to changes in maternal factors/care (Matthews, 2002), which can contribute to the overall effects of prenatal stress on offspring behavior. Previous rat studies indeed reported that stressed mothers spent less time nursing and licking their pups (Muir et al., 1985) and this was associated with depressive-like behavior in the mothers and their offspring, together with an increased response of the offspring HPA axis to stress (Smith et al., 2004). Fostering stressed rat offspring onto unstressed dams prevented their dysregulation of the HPA axis (Maccari et al., 1995) as well as their later anxiogenic profile (Barros et al., 2006) and their brain dopaminergic and glutamatergic alterations (Barros et al., 2004). In mice, although these cross-fostering beneficial effects seemed less evident (Yang et al., 2006) as well as stress-induced deficits in maternal care (Heslin and Coutellier, 2018), long-term neurobehavioral consequences were similarly observed following stressing the mothers or directly the pups (Moles et al., 2004), thus confirming the contribution of post-natal factors in prenatal stress effects. Hence, it is important to control for the effects of stress exposure on maternal care/behaviors, since they may play a key contributing role in the impact on offspring behavior.

**FIGURE 1:**
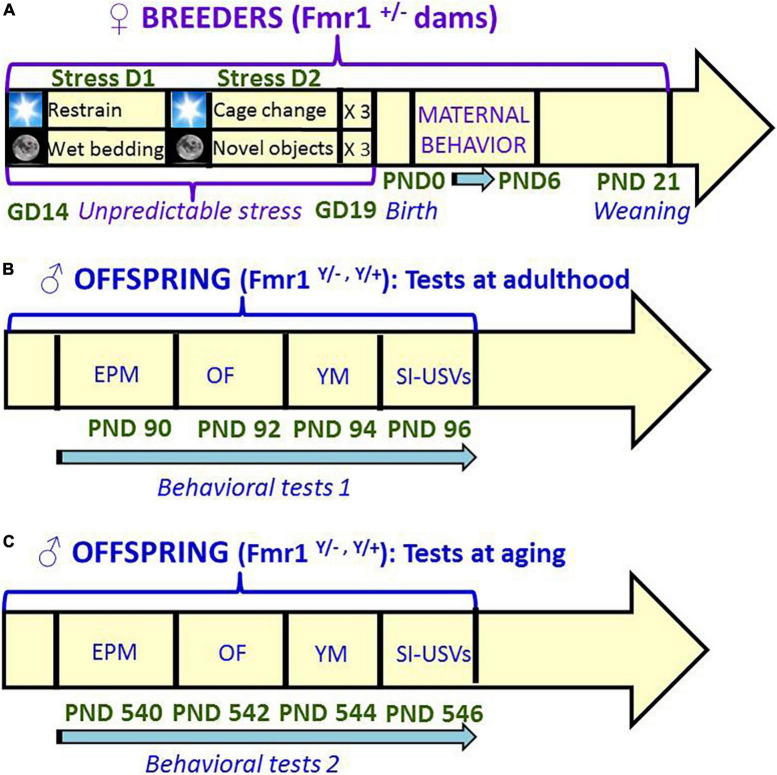
Schematic representation of the timeline of the study. Unpredictable mild stress **(A)** consisted of a 2 day-sequence that was repeated 3 consecutive times during the last week of gestation: on day 1, 3 sessions of 30-min restrain stress during the light phase were followed by overnight housing with wet bedding; on day 2, 3 sessions of sawdust and cage changes during the light phase were followed by overnight housing with novel objects. Control no stress mice were left undisturbed during all pregnancy. Behavioral tests were conducted first at 3 months **(B)** and then at 18 months **(C)** of age, with 48 h interval between each consecutive test. A single cohort of animals was employed for both testing series **(B,C)**. GD, gestational day; PND, postnatal day; EPM, elevated plus maze; OF, open field; YM, Y maze; SI, social interaction; USVs, ultrasonic vocalizations.

Here we evaluated the long-term effects of the exposure to unpredictable chronic mild stress during the last prenatal week on the FXS- and ASD-like behavioral phenotype of the Fmr1-KO model (as schematized in Figure 1). Both FXS and ASD indeed lack any pathological biomarker other than behavioral alterations; hence, the only endpoint with an accepted therapeutic or pathological validity is represented by behavioral symptoms. Fmr1-KO male (hemizygous, -/Y) mice, together with their WT littermates, underwent behavioral tests for anxiety (elevated plus maze) and exploration (open field), spatial memory (Y maze), social interaction and communication (direct social interaction with an adult female) first at adulthood (3 months of age) and then during aging (at 18 months). This long-term behavioral assessment was necessary to evaluate the stability of the stress effects in our Fmr1 mutant mice, concomitantly extending their behavioral characterization during aging, an issue that so far has been mostly neglected. During the first week after birth, maternal behavior was evaluated in stressed and no-stress breeders (Figure 1), in order to control for potential stress effects during the early post-natal phase. The unpredictable chronic mild stress procedure, combining multiple stressors of different nature, was chosen to minimize habituation and avoid pain or nutritional effects (Imbe et al., 2006; Campos et al., 2013), but also because of its high translational validity as a model of early environmental adversity in laboratory rodents (Mineur et al., 2003, 2006a; Willner, 2005). The coincidence of the timing of stress exposure with the last week of gestation of the dams was in line with the majority of previous preclinical studies (reviewed in Weinstock, 2008; Sandi and Haller, 2015), selecting this pregnancy phase to induce long-term neurobehavioral modifications in the offspring, because of its high sensitivity to environmental insults and stressors (Misdrahi et al., 2005; Enayati et al., 2012).

## Materials and methods

### Ethics approval

All experimental procedures were in accordance with the European Communities Council Directive 2010/63/EEC, and approved by local ethical committee (“Comité d’Ethique pour l’experimentation animale de Bordeaux,” CE 50) and the French Ministry (“Ministere de l’enseignement superieur de la recherché et de l’innovation”).

### Breeding and stress procedure

A schematic representation of the experimental plan employed in the study is provided in Figure 1. Twenty adult (12 ± 1 weeks-old) virgin Fmr1 heterozygous (±) females and 10 C57BL/6J adult wild type males [16 weeks-old; purchased from Janvier (Le Genest St Isle, France)] were used as breeders to generate the offspring to be behaviorally tested. C57BL/6JFmr1*^TM^*^1^*^Cgr/Nwu^* (B6) mice were originally obtained from Neuromice.org (Northwestern University) and maintained on the C57BL6/J background.

Two heterozygous females were housed with a WT male for 2 weeks. Males were then removed, while pregnant females (previously identified by the presence of semen in vaginal smears) were single-housed for 1 week before parturition [as done in all our previous studies with Fmr1 mice to avoid the well-known effects of maternal social enrichment on the offspring (Branchi, 2009; D’Amato et al., 2011; Oddi et al., 2015)]. Each half of them was assigned for the last week of pregnancy to one of the following experimental groups: no-stress, i.e., kept undisturbed in their home-cage, or stress, i.e., exposed to the unpredictable stress procedure described below.

The stress procedure included the following sequence of events that was repeated three consecutive times during the last gestational week.

Day 1: 30 min of restrain stress (three times each day during the light phase, with a 4 h-interval) in perforated conical tubes (3 cm in diameter, 11.5 cm long, Becton Dickinson Labware Europe, France), followed by overnight housing with wet bedding (50 ml of water were added to floor sawdust of the home cage at the beginning of the dark phase).

Day 2: multiple sawdust and cage changes (three times each day during the light phase, with a 4 h-interval), followed by overnight housing with novel objects (12 glass black beads, 1.5 cm in diameter were added in the home cage at the beginning of the dark phase).

Pregnant females were exposed to this sequence of events for three times during the last week before parturition: this procedure is known to avoid habituation to stressful stimuli without pain or metabolic effects and is commonly used in rodent studies (e.g., Pardon M. et al., 2000; Pardon M. C. et al., 2000; Negroni et al., 2004; Misdrahi et al., 2005). All breeders included in the study gave birth within 48 h after the last day of exposure to the stress procedure. No alteration in the general health status of stressed breeders emerged at the end of the stress paradigm, based on the daily observation of the animals in their home cage in order to assess both behavioral and physical indicators of welfare (Burkholder et al., 2012). Only litters including males of both genotypes were used for experiments, for a total of 12 litters. They were left undisturbed until weaning of the pups, on post-natal day (PND) 21.

### Assessment of maternal behavior of the breeders

The maternal behavior of a subset of all breeders (six for each stress condition) was observed in their home-cages twice a day for 1 h (at 9.00 a.m. and at 5.00 p.m.) from PND 1 to PND 6, using an instantaneous sampling method (one sampling/2 min, for a total of 30 sampling points/session). The following items were scored as absolute frequencies by an observer who was blind to the stress condition of the breeders (Champagne et al., 2007; Curley et al., 2012; Oddi et al., 2015): (i) nursing postures, including arched-back nursing (the female is in an arched position over the nursing pups) and blanket nursing (the female is lying flat on top of the pups), (ii) non-nursing postures (the female is in contact with the pups, but not nursing, i.e., with no access to the nipples), (iii) licking/grooming of the pups.

### Animals and housing procedures

At 3 weeks of age, all pups were weaned and housed in same-sex groups of 3–5 littermates. On the same day, tail samples were collected for DNA extraction and subsequent PCR assessment of the genotypes as previously described (The Dutch-Belgian Fragile X Consortium, 1994). Mice were then left undisturbed until the beginning of behavioral testing (i.e., at 3 months of age). A single cohort of animals including 39 male (19 WT and 20 Fmr1-KO, *n* = 12 for no stress and 7–8 for stressed conditions) littermates underwent behavioral testing at 3 months. The 34 surviving animals of the same cohort (18 WT and 16 Fmr1-KO, *n* = 10–11 for no stress and 6–7 for stressed conditions) were tested again at 18 months. The sample size was based on our previous work on the effects of prenatal stress in juvenile Fmr1-KO mice (Petroni et al., 2022). Only males were tested, as they are most commonly employed in neurobehavioral studies on FXS because of the higher prevalence of the pathology in the male sex (Lozano et al., 2016) and since Fmr1-KO males show more robust and marked FXS-like phenotypes than mutant females (as reviewed in Pietropaolo and Subashi, 2014).

Stimulus mice used for the direct social interaction test were adult (10 weeks of age) female NMRI mice, as this strain is commonly employed in social studies (Moles and D’Amato, 2000; Moles et al., 2007), especially those using the Fmr1-KO mouse model (Pietropaolo et al., 2011, 2014; Hebert et al., 2014; Oddi et al., 2015; Gaudissard et al., 2017; Gauducheau et al., 2017). This strain is often chosen since it shows high levels of sociability, and it facilitates the behavioral analysis during social encounters with B6 mutants because of its albino phenotype. NMRI mice were purchased from Janvier (Le Genest-Saint-Isle, France), housed in groups of 3–4 per cage and left undisturbed for 2 weeks before being used in behavioral tests.

All animals were housed in polycarbonate standard cages (37 × 21 × 15 cm in size; Tecniplast, Limonest, France), provided with sawdust bedding (SAFE, Augy, France) and a stainless steel wired lid. Food chow (SAFE, Augy, France) and water were provided *ad libitum*. The animals were maintained in a temperature- (22°C) and humidity- (55%) controlled vivarium, under a 12:12 h light–dark cycle (lights on at 7 a.m.).

### Behavioral testing procedures

As described in Figure 1, mice were tested first at three (ADULT group) and then at 18 months (OLD group) of age following the same sequence of tests, each separated by a 48 h-interval. Animals were first subjected to the elevated plus maze to evaluate anxiety-like behavior, followed by the open field test for locomotion and exploration; they were then assessed for spontaneous alternation in the Y-maze, and finally evaluated in a direct social interaction test. The duration of all tests was in line with the most common procedures used in laboratory mice in general (Crawley, 2007) and in Fmr1-KO mice in particular (e.g., Pietropaolo et al., 2011; Pietropaolo and Subashi, 2014; Oddi et al., 2015; Lemaire-Mayo et al., 2017). All behavioral tests were carried out during the light phase of the cycle (between 9 a.m. and 4 p.m.) by an experimenter who was blind to the group assignment of the subjects. All mice were habituated to the experimental room for at least 30 min before the beginning of each behavioral test.

#### Elevated plus maze

The maze described in detail elsewhere (Pietropaolo and Crusio, 2009; Pietropaolo et al., 2011) was placed 55 cm above floor level, in a quiet testing room with diffuse dim lighting (55 lux in the maze center). A digital camera was mounted above the maze, and images were transmitted to a PC running the Ethovision (Version 13, Noldus Technology, Netherlands) tracking system. To begin a trial, the mouse was gently placed in the central square with its head facing one of the open arms and allowed to explore freely for 5 min. We measured the percent time in open arms as [time_(open arms)_/time_(open + closed arms)_] × 100.

#### Open field

The open field consisted of a white opaque plastic arena (42 × 26 × 15 cm) under dim lighting conditions (55 lux). Each mouse was placed in the center of the arena and allowed to freely explore it for 10 min. Locomotor habituation, requiring longer testing sessions, was not assessed since it is known to be unaltered in Fmr1-KO adult mice (e.g., Pietropaolo et al., 2011, 2014; see also Pietropaolo and Subashi, 2014 for a review). Automated tracking of the videos obtained from a camera mounted above the open field was performed by Ethovision to analyze the distance traveled.

#### Y maze

A gray plastic Y-maze (each arm measuring 8 × 42 × 15 cm, 120° spaced) was placed on a table 80 cm high, in a room with extramaze cues on the walls. For the habituation phase, each mouse was introduced to the end of one maze arm and allowed to explore two arms for 5 min, while the access to the third arm was blocked by a transparent plastic door. After an interval of 10 min in a waiting cage, the testing phase began: the door of the blocked arm was removed and the mouse was allowed to explore all three arms for 2 min. Allocations of the start and blocked arms were counterbalanced within experimental groups. Time spent in each arm during the habituation and testing phases was scored by Ethovision through automatic tracking of the videos collected from a camera mounted above the maze center. We measured the percent alternation rate as [time_(novel arm)_/time_(all arms)_] × 100.

#### Social interaction and ultrasonic communication

All mice were tested in a 33 × 15 × 14 cm plastic cage with 3 cm of sawdust and a metal flat cover. Male experimental subjects were habituated to this apparatus for 30 min prior to testing; an unfamiliar stimulus female mouse (an adult NMRI female, different for each tested male) was then introduced into the testing cage and left there for 3 min. Previous studies have shown that in these experimental settings USVs are emitted only by the male mouse in the male-female interaction (Whitney et al., 1973; Maggio and Whitney, 1985). Furthermore, all spectrograms obtained here were additionally inspected to exclude the presence of “double calls,” i.e., overlapping in their timing, but with different, non-harmonic, characteristics (e.g., different peak and mean frequency). These calls would in fact suggest the concomitant emission of USVs by the two interacting subjects during testing.

Testing sessions were recorded by a camera placed on the side of the cage and videos analyzed with Observer XT (Noldus, Netherlands). One observer who was unaware of the genotype and stress conditions of the animals scored the behavior of the test male mice, quantifying the time spent performing affiliative behaviors (Pietropaolo et al., 2011, 2014; Oddi et al., 2015; Gaudissard et al., 2017; Gauducheau et al., 2017), i.e., sniffing the head and the snout of the partner, its anogenital region, or any other part of the body; contact with partner through traversing the partner’s body by crawling over/under from one side to the other or allogrooming. Non-social activities were also measured: rearing (standing on the hind limbs sometimes with the forelimbs against the walls of the cage); digging; self-grooming (the animal licks and mouths its own fur).

**FIGURE 2:**
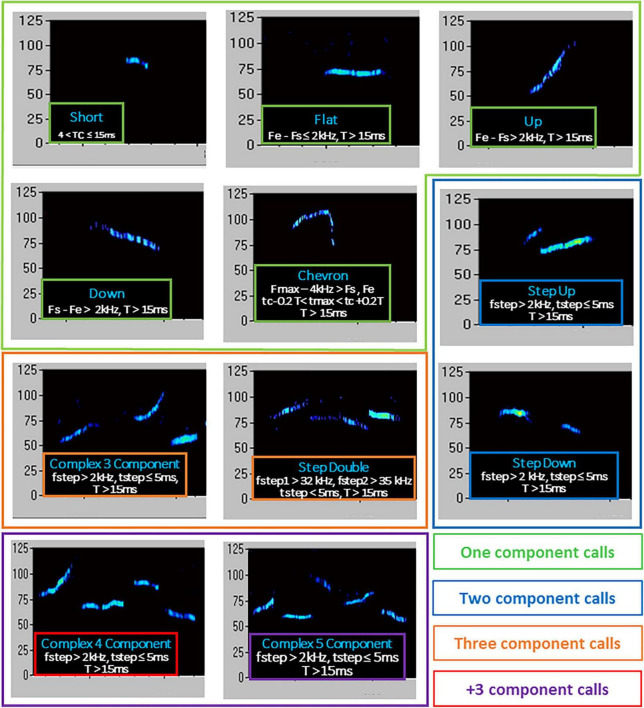
Type of ultrasonic calls evaluated in the study. T, duration of call; Fs, start frequency; Fe, end frequency; Fmax, highest frequency; Fmin, lowest frequency; fstep, frequency step; tmax, time of max frequency; tmin, time of min frequency; tc, time at center of call. Call types were automatically classified using the software Sonotrack, based on the parameters described here. Definitions of call types were mutually exclusive. Overlap of components was removed when more than 70% to prevent wrong call durations. Short gaps between components in both frequency (≤6 kHz) and time (≤5 ms) were interpolated (gaps can be caused by changes in microphone sensitivity or direction of vocalization). The calls classified as “complex 3 component” and “+3 component” were summed up into a “total complex” category.

An ultrasonic microphone UltraSoundGate Condenser Microphone CM 16 (Avisoft Bioacoustics, Berlin, Germany) was mounted 2 cm above the cover of the testing cage; it was connected *via* an UltraSoundGate 116 USB audio device (Avisoft Bioacoustics) to a personal computer, where acoustic data were recorded with a sampling rate of 250 kHz in 16-bit format by Avisoft Recorder (version 2.97; Avisoft Bioacoustics). Recordings were then transferred to the Sonotrack Call Classification Software (version 1.4.7, Metris B.V., Netherlands). This software fully automatically recognizes up different USV types and also calculates quantitative parameters including the total number and mean duration of the calls. Based on previous literature on call types (Scattoni et al., 2011; Premoli et al., 2019; Caruso et al., 2020), the following USV types were selected for automatic recognition in our dataset: Short, Flat, (Ramp) Up, (Ramp) Down, Chevron, Step-Up, Step-Down, Step-Double (Split), Complex-3, Complex-4, Complex-5, Complex-5+. Their characteristics are described in detail in Figure 2.

The estrus phase of female NMRI stimulus mice was assessed by analysis of vaginal smears (Caligioni, 2009) performed on the testing day. Stimulus NMRI females were approximately half in diestrus and half in estrus phases, and their assignment to social encounters was equally distributed between experimental groups.

### Statistical analysis

Data from maternal behavior of the breeders were analyzed with an ANOVA with stress as the between-subject factor and 2-day blocks as the within-subject variable. All data from the offspring behaviors were separately analyzed in adults and old mice. This was due to differences in the number of mice present at the two testing points (38 adults versus 34 old mice), because of the loss of some animals due to aging.

Data from each age were then analyzed with a 2 × 2 ANOVA with genotype and stress as the between subject factors (Table 1). Alternation rates from the Y-maze test were instead analyzed for differences from the chance level (with a *t*-test), in line with previous studies (Baudonnat et al., 2011; Vandesquille et al., 2013). This analysis served to assess whether the animals showed a significant arm preference in the first place, and thus allows checking whether the behavioral test worked as expected, at least in control mice. USV call types were expressed as percent proportion of total USVs: their measures are therefore not independent, since they all sum up to the total number of USVs for each experimental group. Therefore, no correction for multiple comparisons was needed, in line with a multitude of studies assessing multiple ultrasonic call categories in mouse models [e.g., the seminal papers on mouse call types in the BTBR model (Scattoni et al., 2011)], in the Fmr1-KO pups (Nolan et al., 2020) and methodological articles on Shank-KO mice (de Chaumont et al., 2021). The statistical effect of the estrous phase of the stimulus female was assessed on social behaviors and ultrasonic communication by a 2 × 2 × 2 (genotype × stress × estrous) ANOVA of all variables measured during the social interaction test (Table 2).

**TABLE 1:** Statistical outcome of the genotype × stress ANOVA of all variables related to offspring behaviors in adult and old mice.

Test	Variable	Adults									Old								
		Genotype			Stress			Genotype × Stress			Genotype			Stress			Genotype × Stress		
		*DF*	*F-value*	*P-value*	*DF*	*F-value*	*P-value*	*DF*	*F-value*	*P-value*	*DF*	*F-value*	*P-value*	*DF*	*F-value*	*P-value*	*DF*	*F-value*	*P-value*
***EPM***	*% time open arm*	1–33	1.4	0.4	1–33	13.64	**0.0008**	1–33	0.031	0.86	1–26	17.7	**0.0003**	1–26	0.11	0.74	1–26	4.68	**0.04**
***OF***	*Total distance*	1–31	7.11	**0.01**	1–31	5.47	**0.02**	1–31	4.3	**0.04**	1–27	3.47	0.07	1–27	5.58	**0.02**	1–27	1.06	0.31
***SI***	*Time affiliation*	1–31	5.66	**0.02**	1–31	2.01	0.16	1–31	0.19	0.66	1–26	2.69	0.11	1–26	0.5	0.82	1–26	4.23	**0.049**
	*nb USVs*	1–34	1.36	0.25	1–34	0.38	0.54	1–34	0.03	0.87	1–29	0.28	0.6	1–29	1.92	0.17	1–29	0.02	0.88
	*dur USVs*	1–34	0.23	0.63	1–34	0.1	0.75	1–34	1.69	0.2	1–30	2.06	0.16	1–30	3.44	0.07	1–30	0.06	0.81
	*% short calls*	1–34	0.05	0.82	1–34	0.01	0.91	1–34	0.44	0.51	1–29	0.67	0.42	1–29	0.17	0.68	1–29	0.16	0.69
	*% flat calls*	1–34	0.7	0.41	1–34	0.41	0.52	1–30	0.2	0.65	1–30	1.19	0.28	1–30	5.11	**0.03**	1–30	0.11	0.75
	*% up calls*	1–34	1.51	0.23	1–34	1.03	0.31	1–34	0.15	0.7	1–29	0.36	0.55	1–29	0.14	0.7	1–29	0.24	0.63
	*% down calls*	1–34	1.43	0.24	1–34	0.64	0.43	1–34	0.05	0.82	1–29	1.9	0.18	1–29	0.22	0.64	1–29	1.22	0.28
	*% chevron calls*	1–34	0.34	0.56	1–34	0.87	0.36	1–34	0.69	0.41	1–29	0.04	0.83	1–29	0.53	0.47	1–29	0.32	0.57
	*% step down calls*	1–34	0.9	0.35	1–34	0.2	0.66	1–34	0.68	0.41	1–29	1.6	0.21	1–29	0.48	0.5	1–29	0.03	0.86
	*% step up calls*	1–34	0.04	0.84	1–34	0.2	0.65	1–34	0.61	0.44	1–29	0.46	0.5	1–29	0.32	0.57	1–29	1.3	0.26
	*% step double calls*	1–34	0.93	0.34	1–34	0.01	0.91	1–34	1.35	0.25	1–29	1.92	0.17	1–29	0.2	0.66	1–29	0.76	0.4
	*% complex tot calls*	1–34	2.36	0.13	1–34	0.44	0.51	1–34	3.84	0.06	1–30	3.85	0.06	1–30	6.97	**0.01**	1–30	0.16	0.7

EPM, elevated plus maze; OF, open field; SI, social interaction; Nb, number; Dur, mean duration; USVs, ultrasonic vocalizations. Dark gray highlights and bold characters indicate significant values (*p* < 0.05); when these values refer to a genotype × stress interaction *post-hoc* Tukey–Kramer tests were conducted. Light gray highlights indicate non-significant tendencies (0.05 ≤ *p* < 1). Slight differences in the exact number of mice between tests or variables are due to technical reasons (e.g., loss of behavioral video recordings, animals falling from the elevated plus maze) or to the exclusion of outliers (using Grubbs’ ESD test adapted for small sample size). The exact *n* for each variable is provided in the corresponding figures. Data from the Y-maze test were instead analyzed by *t*-tests versus the chance level.

**TABLE 2:** Statistical outcome of the stimulus estrous × genotype × stress ANOVA of all variables measured during the social interaction (SI) test.

Adults												Old											
Estrous			Estrous × Genotype			Estrous × Stress			Estrous × Genotype × Stress			Estrous			Estrous × Genotype			Estrous × Stress			Estrous × Genotype × Stress		
							
DF	*F*-value	*P*-value	DF	*F*-value	*P*-value	DF	*F*-value	*P*-value	DF	*F*-value	*P*-value	DF	*F*-value	*P*-value	DF	*F*-value	*P*-value	DF	*F*-value	*P*-value	DF	*F*-value	*P*-value
1–27	0.24	0.63	1–27	0.18	0.68	1–27	0.41	0.53	1–27	0.03	0.86	1–22	0.44	0.51	1–22	0.02	0.88	1–22	0.92	0.35	1–22	0.13	0.72
1–30	0.003	0.96	1–30	0.23	0.63	1–30	0.002	0.97	1–30	0.05	0.82	1–25	0.01	0.92	1–25	0.07	0.79	1–25	0.03	0.87	1–25	0.39	0.54
1–30	0.11	0.74	1–30	0.01	0.92	1–30	0.28	0.6	1–30	0.12	0.73	1–25	0.56	0.46	1–25	0.54	0.47	1–25	0.27	0.61	1–25	0.23	0.63
1–30	0.07	0.79	1–30	0.27	0.61	1–30	0.15	0.7	1–30	0.5	0.49	1–25	2.2	0.15	1–25	0.9	0.35	1–25	0.42	0.52	1–25	0.17	0.68
1–30	0.06	0.81	1–30	2.45	0.13	1–30	1.22	0.28	1–30	0.32	0.57	1–25	2.29	0.14	1–25	0.15	0.7	1–25	0.09	0.76	1–25	0.27	0.61
1–30	0.17	0.68	1–30	0.8	0.38	1–30	1.99	1.7	1–30	1.05	0.31	1–25	0.16	0.69	1–25	0.16	0.69	1–25	0.16	0.69	1–25	0.006	0.94
1–30	0.007	0.93	1–30	0.27	0.61	1–30	0.007	0.93	1–30	0.07	0.8	1–25	0.65	0.43	1–25	3.29	0.08	1–25	0.02	0.89	1–25	0.57	0.46
1–30	0.003	0.95	1–30	0.09	0.77	1–30	0.02	0.89	1–30	0.7	0.41	1–25	0.43	0.52	1–25	1.81	0.19	1–25	0.73	0.4	1–25	1.04	0.32
1–30	0.51	0.48	1–30	0.006	0.94	1–30	0.2	0.66	1–30	1.35	0.25	1–25	0.005	0.94	1–25	1.95	0.17	1–25	2.56	0.12	1–25	0.07	0.79
1–30	0.83	0.37	1–30	0.09	0.77	1–30	0.29	0.59	1–30	0.68	0.42	1–25	0.6	0.44	1–25	3.29	0.08	1–25	0.59	0.45	1–25	0.001	0.98
1–30	<0.001	0.99	1–30	0.12	0.73	1–30	1.84	0.18	1–30	0.01	0.91	1–25	0.08	0.78	1–25	0.41	0.52	1–25	0.74	0.4	1–25	0.85	0.37
1–30	0.1	0.75	1–30	1.48	0.23	1–30	0.06	0.81	1–30	1.05	0.31	1–25	0.21	0.65	1–25	1.12	0.3	1–25	1.02	0.32	1–25	0.28	0.6

NMRI adult stimulus females used during the social interaction (SI) test of the Fmr1 offspring were either in estrous or diestrous phase. The allocation of each stimulus female was balanced across experimental groups so that half of each genotype × stress group encountered either a diestrous or estrous female. Different batches of stimulus female were used for testing at the two ages. No significant effect of estrous or of its interactions was found by the 2 × 2 × 2 (estrous × genotype × stress) ANOVA of all social and USV-related parameters. Slight differences in the exact number of mice (provided in the corresponding figures) are due to technical reasons (e.g., loss of behavioral video recordings) or to the exclusion of outliers (using Grubbs’ ESD test adapted for small sample size). Nb, number; Dur, mean duration; USVs, ultrasonic vocalizations.

*Post-hoc* comparisons were performed using Tuckey–Kramer test when a significant interaction was detected. Analyses were performed using the software Statview and SPSS and α was set at 0.05. Results are expressed as mean ± SEM. Slight differences in the exact number of mice between tests on the offspring are due to technical reasons (e.g., loss of behavioral video recordings, animals falling from the elevated plus maze or not exploring during the habituation phase of the Y-maze) or to the exclusion of outliers (using Grubbs’ ESD test adapted for small sample size); the precise sample size for each variable is provided in each figure.

## Results

### Maternal behavior of the breeders

**FIGURE 3:**
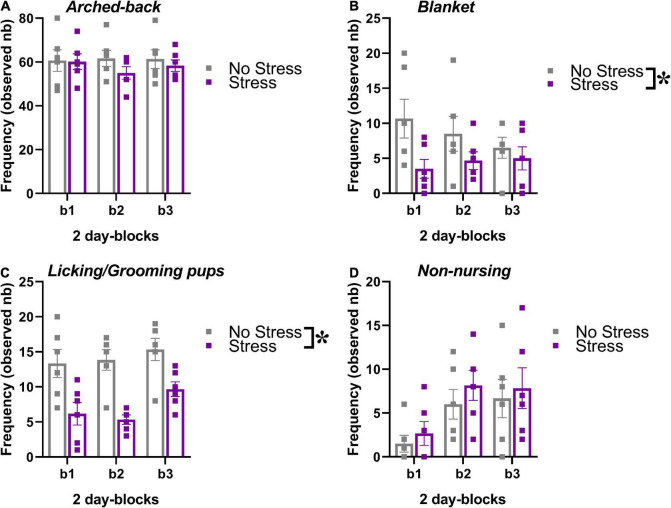
Effects of prenatal stress on the maternal behavior of the breeders used to generate the offspring tested in the study. The maternal behavior of a subset of all breeders (*n* = 6 for each stress condition) was observed in their home-cages twice a day for 1 h (at 9.00 a.m. and at 5.00 p.m.) from PND 1 to PND 6, using an instantaneous sampling method (one sampling/2 min). The following items were scored as absolute frequencies by an observer who was blind to the experimental conditions of the breeders (Champagne et al., 2007; Curley et al., 2012; Oddi et al., 2015): (i) nursing postures, including arched-back nursing (the female is in an arched position over the nursing pups, **A**) and blanket (the female is lying flat on top of the pups, **B**), (ii) licking/grooming of the pups **(C)**, (iii) non-nursing postures (the female is in contact with the pups, but not nursing, i.e., with no access to the nipples, **D**). All behaviors were illustrated across 2-day blocks (b1, b2, b3). *p < 0.05 from the ANOVA with stress as the between-subject factor and 2-day blocks as the within-subject variable leading to a significant main effect of stress **(B,C)**. Data are expressed as mean ± SEM.

The behavior of the dams in their home-cage was scored during the first post-natal week of the pups, i.e., between PND 1 and 6. No effect of time was detected on the occurrence of nursing postures (arched-back and blanket) or grooming/licking of the pups [Figures 3A–C, main effect of 2-day blocks: *F*(2,20) = 0.47, 0.23, 1.83; *p* = 0.63, 0.79, 0.19], but a significant increase across days was found on the frequency of non-nursing postures [main effect of 2-day blocks: *F*(2,20) = 6.03, *p* < 0.01; Figure 3D]. Compared to no-stressed controls, stressed dams were less engaged in nursing postures, in particular in the blanket posture and in licking/grooming of their pups [stress effect, respectively: *F*(1,10) = 7.86 and 128.40, *p* < 0.05 and 0.0001; Figures 3B,C]. No effect of stress was found on arched back posture and non-nursing postures [Figures 3A,C, main effect of stress: *F*(1,10) = 0.54, 0.92; *p* = 0.48,0.37]. The interaction stress × 2-day blocks was not significant for all 4 variables [*F*(2,20) = 0.97,1.05, 0.34, 0.06; *p* = 0.40, 0.37, 0.71, 0.94].

### Offspring behaviors

Stress effects on selected behaviors differed according to the age of testing and sometimes depending on the genotype, as it was clearly shown by the ANOVAs’ results described below as well as in Table 1.

#### Elevated plus maze

**FIGURE 4:**
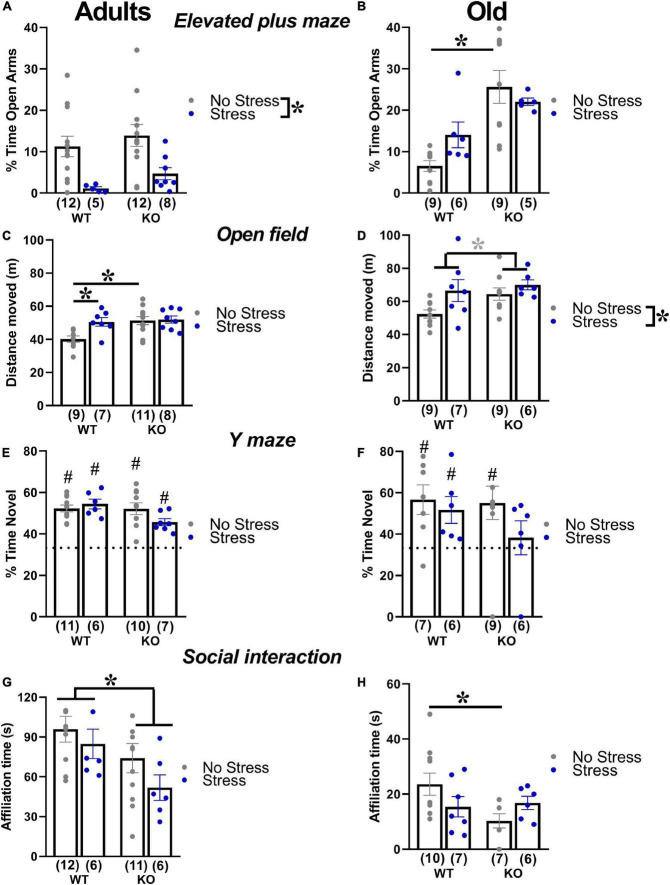
Behavioral effects of prenatal stress in adults (3 months) and old (18 months) mice. Anxiety levels were investigated with the elevated plus maze test **(A,B)**. Locomotion was assessed in the open field **(C,D)**, while spontaneous alternation was evaluated in the Y maze **(E,F)**. Social interaction was measured during a 3-min encounter with an adult NMRI WT female **(G,H)**. *p < 0.05 from the 2 × 2 ANOVA at each age with genotype and stress as the between subject factors, leading to significant main effects of stress **(A,D)**, genotype **(G)** or stress x genotype interaction followed by *post-hoc* tests **(B,C,H)**. *p = 0.07 from the same ANOVA leading to a nearly significant main effect of stress **(D)**. ^#^*p* < 0.05 from the *t*-test versus chance level [indicated by dotted line in panels **(E,F)**] conducted in each experimental group. N for each group are indicated as (N) in each graph. Data are expressed as mean ± SEM.

In adult mice, stress enhanced anxiety levels, that is, it reduced the percent time spent in the open arms in animals of both genotypes [stress effect: *F*(1,33) = 13.64, *p* < 0.001; Figure 4A]. In old mice, KO animals showed lower anxiety levels than WTs, but only under no stress condition [genotype X stress interaction: *F*(1,26) = 4.68, *p* < 0.05; WT-no stress versus KO-no stress, *post-hoc*: *p* < 0.05; Figure 4B and Table 1].

#### Open field

In adults, KO mice were more active than their WT littermates and this effect was detected only in no-stress conditions; furthermore, stress increased locomotor activity in WT mice [interaction genotype X stress *F*(1,31) = 4.03, *p* < 0.05; WT-no stress versus KO-no stress and WT-stress, *post-hoc*: *p* < 0.05; Figure 4C and Table 1]. Old KO mice also tended to be hyperactive compared to their WT littermates, [main effect of genotype *F*(1,27) = 3.47, *p* = 0.07; Figure 4D]. At this age, stress induced an overall increase in locomotor activity [main effect of stress *F*(1,27) = 5.58, *p* < 0.05; Figure 4D].

#### Y maze

In adults, all experimental groups showed alternation rates significantly higher than the chance level (*t*-tests versus the chance level, *p* < 0.05; Figure 4E). Aged mice displayed alternation rates significantly higher than the chance level (*t*-tests versus the chance level, *p* < 0.05; Figure 4F), with the exception of the KO-stressed group (*t*-test versus the chance level, *p* = 0.57; Figure 4F).

#### Social interaction

In adults, KO mice were less social than their WT littermates, spending less time sniffing the stimulus female, and this effect was observed in both stress conditions [genotype effect: *F*(1,31) = 5.66, *p* < 0.05; Figure 4G and Table 1]. Old KO mice were less social than their WT littermates, spending less time sniffing the conspecific, but this effect was observed only in no stress conditions [interaction genotype × stress: *F*(1,26) = 4.23, *p* < 0.05; *post-hoc*: WT-no stress versus KO-no stress; Figure 4H].

#### Ultrasonic communication

**FIGURE 5:**
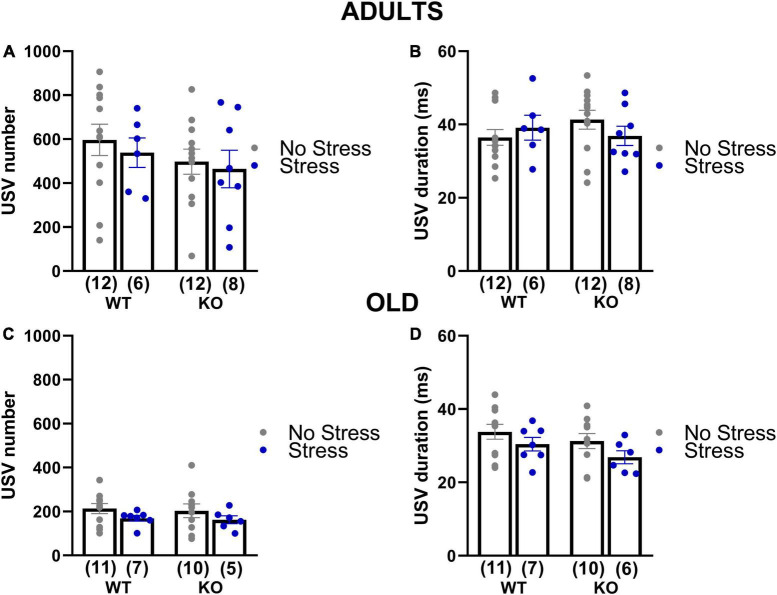
Quantitative analysis of the ultrasonic vocalizations (USVs) emitted during the social interaction test in adult and old mice (3 and 18 months of age). USVs were recorded during the direct social interaction test. Male experimental subjects were habituated to the testing cage for 30 min prior to testing; an unfamiliar adult stimulus female mouse was then introduced and left there for 3 min. Previous studies have shown that in these experimental settings USVs are emitted only by the male mouse in the male-female interaction (Whitney et al., 1973; Maggio and Whitney, 1985). The number **(A,C)** and mean duration **(B,D)** of USVs were automatically measured using the software Sonotrack. Data are expressed as mean ± SEM. N for each group are indicated as (N) in each graph.

In adults and old mice, no significant effect of genotype or stress was found on the total number of the USVs or their mean duration (all effects and their interaction in adults and old, n.s.; Figure 5 and Table 1), except a non-significant tendency of old stressed mice to emit shorter calls [stress effect on the mean duration in old mice: *F*(1,30) = 3.44, *p* = 0.07; Figure 5D].

**FIGURE 6:**
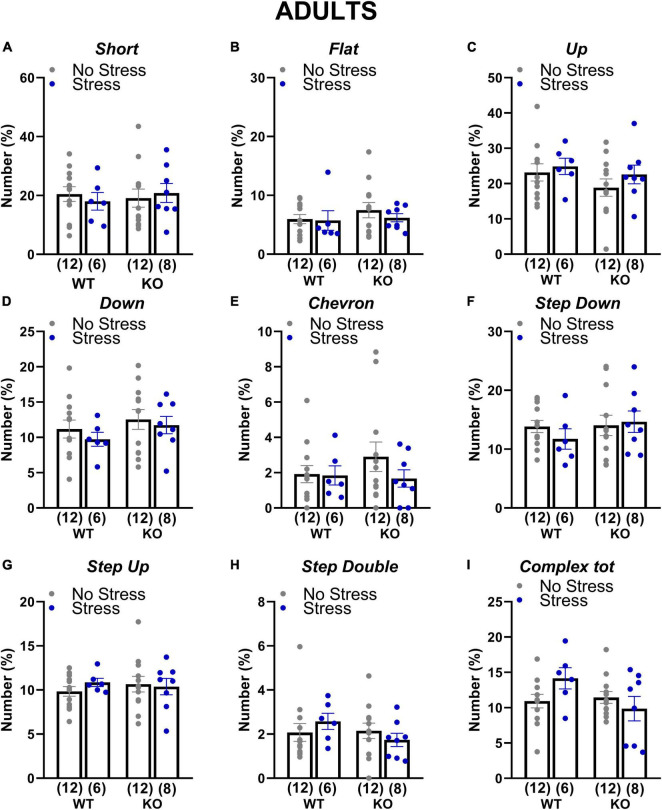
Qualitative analysis of the ultrasonic vocalizations (USVs) emitted during the social interaction test in adult mice. Ultrasonic calls were automatically categorized as described in detail in Figure 2 **(A–H)**. The “complex tot” category **(I)** included all complex calls with more than 3 components. Data are expressed as mean ± SEM. N for each group are indicated as (N) in each graph.

**FIGURE 7:**
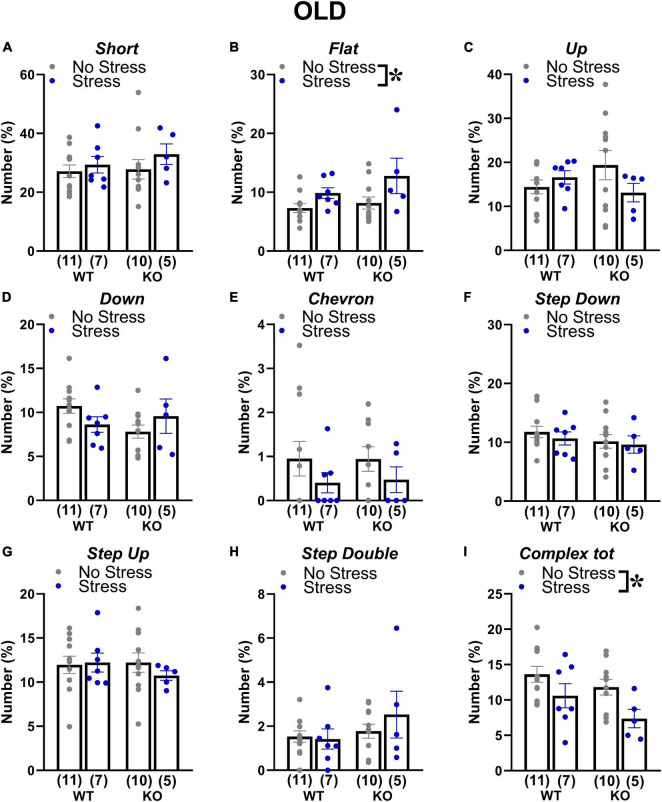
Qualitative analysis of the ultrasonic vocalizations (USVs) emitted during the social interaction test in old mice. Ultrasonic calls were automatically classified in the categories described in detail in Figure 2 **(A–H)**. The “complex tot” category **(I)** contained all complex calls with more than 3 components. Data are expressed as mean ± SEM. *p < 0.05 from the 2 × 2 ANOVA at old age with genotype and stress as the between subject factors, leading to a significant main effect of stress **(B,I)**. N for each group are indicated as (N) in each graph.

At adulthood only a slight difference among experimental groups emerged in the proportion of complex calls, but failed to reach statistical significance [interaction genotype × stress: *F*(1,34) = 3.84; *p* = 0.06; stress effect in separate ANOVA in WT mice: *F*(1,16) = 3.52, *p* = 0.08; in KOs: *F*(1,18) = 0.84, *p* = 0.37; Figure 6]. In old mice, stress increased the percentage of flat calls [stress effect: *F*(1,30) = 5.11, *p* < 0.05; Figure 7] while it reduced the percentage of complex calls with 3 or more components [stress effect: *F*(1,30) = 6.97, *p* < 0.05; Figure 7]. These complex calls tended also to be decreased in KO mice, although this effect failed to reach statistical significance [genotype effect: *F*(1,30) = 3.85, *p* = 0.06; Figure 7 and Table 1].

The estrous cycle of the stimulus female (estrous versus diestrous, *n* = 3–6 for each condition) did not affect the social and ultrasonic behaviors of mice at both ages (Table 2). This lack of effect could be due to the small sample size, although this was similar to the one used in previous studies on estrous cycle and ultrasonic communication in laboratory mice (Premoli et al., 2022).

## Discussion

Our findings demonstrated that prenatal exposure to chronic unpredictable stress is able to induce selected long-term behavioral effects in the Fmr1-KO mouse model for FXS and ASD. The data obtained in the present study also highlighted the critical relevance of the complex interactions occurring between genetic and environmental factors, suggesting subtle age-specific differences in the impact of prenatal stress on the behavioral phenotype of both WT and Fmr1 mutant mice.

First of all, our results confirmed and extended most of the previous reports on the adult behavioral phenotype of Fmr1-KO mice (reviewed in Pietropaolo and Subashi, 2014). Adult Fmr1 mutants showed an expected hyperactivity in the open field (The Dutch-Belgian Fragile X Consortium, 1994; Mineur et al., 2002; Restivo et al., 2005; Spencer et al., 2005, 2011; Eadie et al., 2009; Pietropaolo et al., 2011, 2014; Hebert et al., 2014; Oddi et al., 2015) and reduced social interaction (Mineur et al., 2006b; McNaughton et al., 2008; Dahlhaus and El-Husseini, 2010; Hebert et al., 2014; Pietropaolo et al., 2014; Oddi et al., 2015), in the absence of deficits in spontaneous alternation, anxiety or ultrasonic communication. This behavioral phenotype was replicated at 18 months of age, but it was accompanied by reduced levels of anxiety that were displayed by Fmr1-KO old mutants only. Hence, our findings suggest the adult behavioral phenotype of Fmr1 mutants may become slightly more varied with age, in line with what was previously described for non-behavioral effects of the Fmr1 deletion (The Dutch-Belgian Fragile X Consortium, 1994). Our results, providing for the first time a characterization of the behavioral phenotype of the Fmr1-KO mouse model at an advanced age, support the relevance of aging as a sensitive period for the effects of genetic insults, also in the context of neurodevelopmental disorders.

The absence of memory deficits in the Y maze in no-stress mutants both at adulthood and at aging was partially surprising, since we previously described a spontaneous alternation deficit already at 3 months of age (Hebert et al., 2014; Oddi et al., 2015; Lemaire-Mayo et al., 2017). Nonetheless, other previous reports failed to detect a clear deficit in spontaneous alternation in Fmr1-KO mice (e.g., Pietropaolo et al., 2011), thus suggesting that this cognitive alteration (as others in the spatial memory domain) may not be a robust and highly replicable phenotype of this model (Pietropaolo and Subashi, 2014). Our findings here from old animals, combined with previous ones from juvenile Fmr1-KO mice (Petroni et al., 2022), therefore suggest that prenatal stress exposure may represent a “second hit” in the context of gene-environment interactions that is necessary to induce Y maze deficits in this mouse model, although only at specific ages. Similarly, an inconsistent anxiety-like profile of adult Fmr1 mutants was previously described in the elevated plus maze, in line with the one observed here, i.e., more often characterized by unaltered (e.g., Mineur et al., 2002; Nielsen et al., 2002; Zhao et al., 2005; Pietropaolo et al., 2011) or reduced (e.g., de Diego-Otero et al., 2009; Eadie et al., 2009; Liu et al., 2011; Qin et al., 2011; Heulens et al., 2012; Jung et al., 2012; Hebert et al., 2014) anxiety levels in this test. Previous studies have interpreted the reduced anxiety levels displayed by Fmr1-KO mice as a consequence of their altered neurogenesis in the ventral hippocampus and/or reduced corticosterone response to acute stressors (Eadie et al., 2009). In contrast to the inconsistent anxiety phenotype in the elevated plus maze, increased levels of social anxiety appear as a robust behavioral feature of the Fmr1-KO mouse model (see Kat et al., 2022 for a critical review), as confirmed here in the social interaction test showing reduced social investigation in mutants. Our findings on social anxiety are in agreement with previous studies on this mouse line, using direct and indirect measures of social approach/avoidance (e.g., Spencer et al., 2005; McNaughton et al., 2008). The lack of significant quantitative communication deficits in our adult and old control KO mice (Figure 5) was in line with previous reports in FXS patients (Borghgraef et al., 1987) and in this animal model (Pietropaolo et al., 2011; Oddi et al., 2015; Belagodu et al., 2016; Hodges et al., 2017; Lemaire-Mayo et al., 2017). We instead described for the first time a subtle qualitative USV alteration, i.e., a tendency to a reduction in the number of complex calls (consisting of more than 3 components) in Fmr1-KO old mutants (Figure 7I). This limited complexity of the communication phenotype of Fmr1-KOs may agree, although through a tendency that failed to reach statistical significance, with previous analyses of communication abnormalities in FXS mice (Belagodu et al., 2016) and patients (Largo and Schinzel, 1985; Belser and Sudhalter, 2001).

The behavioral phenotype of Fmr1-KO mice was partially modulated by the prenatal exposure to stress that was indeed able to interact with the Fmr1 genetic mutation. More specifically, stress induced, although selectively in old Fmr1-KO mice, the emergence of a deficit in spatial memory in the Y maze: this was evident only at 18 months when the performance of KO-stressed mice was indistinguishable from the chance level (Figure 4F). A similar gene-environment interaction was observed in our previous study in juvenile Fmr1-KO male mice (Petroni et al., 2022), where stress was found to be necessary to generate a robust alteration in spontaneous alternation in these mutants. Interestingly, other interactions between stress exposure and Fmr1 mutation were detected, but this time they were due to the higher behavioral sensitivity of WT mice to stress. Indeed, certain Fmr1-KO phenotypes, such as hyperactivity (at adulthood), reduced anxiety and social interaction (only in older mice), “disappeared” in stress conditions, mainly because of the selective behavioral effects of stress in WT mice, i.e., rendering them “more similar” to their KOs littermates (Figure 4). Stress also induced several age-specific behavioral effects in mice of both genotypes: at adulthood it enhanced anxiety levels and locomotor activity (Figure 4), while at aging it induced hyperactivity (Figure 4), and reduced the complexity of ultrasonic calls (Figure 7). These effects of prenatal stress were mostly in agreement with previous reports in WT rodents (reviewed in Weinstock, 2008; Sandi and Haller, 2015), describing stress-induced reduction of social interaction, increase in anxiety and locomotion. The effects of stress on the complexity of the calls confirmed the role of this qualitative parameter as a sensitive marker of aversive environmental/genetic factors, as observed for the phenotype of Fmr1-KO mice. Future playback studies specifically addressing the communicative value of complex calls may shed light on the precise meaning of this category of USVs in adult mouse social interactions.

The behavioral long-term effects of stress evidenced by our study in mice of both genotypes could be explained by a variety of neurobiological factors. One possible explanation links the behavioral phenotype of stressed animals to their alterations in the development and formation of corticostriatal and corticolimbic pathways, associated with long-term abnormalities of their glutamatergic and dopaminergic tone in different brain regions (Berger et al., 2002). Additional possible interpretations point out the role of stress-induced alterations in vasopressin and oxytocin functionality, in hypothalamic and limbic brain regions (Takayanagi and Onaka, 2021), as well as of the abnormal excitatory/inhibitory (E/I) imbalance or dendritic spine abnormalities observed in the rodent stressed brain (Sandi and Haller, 2015; Marchisella et al., 2021). In particular, future work should focus on the effects of prenatal stress on spine density and functionality in cortical and hippocampal areas, with a particular focus on the prefrontal cortex, based on our data from the Y-maze here and in juveniles (Petroni et al., 2022). Indeed, a pathological phenotype of FXS described in both pre-clinical and clinical settings consists of the retention of abnormally elongated premature spines, related to abnormal synaptic development (Comery et al., 1997; Dolan et al., 2013; Jawaid et al., 2018; Faust et al., 2021).

Beside these considerations, our data highlight the relevance of the early timing of stress exposure: the comparison with previous findings obtained from Fmr1-KO mice with post-natal stress (Qin et al., 2011; Lemaire-Mayo et al., 2017) indeed suggests a more marked impact of the pre-natal stressful experience, with more pronounced and varied behavioral effects; anxiety levels were for example enhanced here in KO stressed mice, while they were unaltered following post-natal stress exposure (Qin et al., 2011; Lemaire-Mayo et al., 2017). This stronger impact of pre-natal versus post-natal (adult) exposure could be due to the direct higher sensitivity to stress of certain brain circuits during the pre-natal phase and/or to some indirect effects of stress on early post-natal development (Matthews, 2002). Here we demonstrated that stressed mothers performed less licking/grooming of the pups and spent less time nursing (Figure 3), in agreement with previous studies on rats (Muir et al., 1985; Maccari et al., 1995; Smith et al., 2004). Nonetheless, previous studies observed long-term behavioral consequences of prenatal stress in mice of different strains, also in the absence of altered maternal care (Heslin and Coutellier, 2018): it is therefore possible that alterations in maternal factors may contribute, but not fully account for the stress effects illustrated by our results.

A characteristic of our study that may be considered as a potential limitation lies in the exclusive focus on stress long-term effects, including only adult and older mice. This could seem as a limitation in its translational value, since most human studies on prenatal stress and the risk of developing ASD and other neurodevelopmental disorders focus instead on pediatric populations. Indeed, future studies are warranted to investigate the effects of prenatal stress in Fmr1-KO mice during development, for example evaluating spine alterations in the prefrontal cortex and other brain areas, as mentioned before. Nonetheless, testing Fmr1-KO mice from adulthood onward has still a translational value in itself, since the pathological phenotypes of FXS and ASD are persistent in older patients. Here, our main aim was to evaluate whether the effects of prenatal stress we observed before in juvenile mutants in our previous study on an independent cohort of mice (Petroni et al., 2022) could be confirmed later on, i.e., in adult and old animals. Interestingly, we could detect the same effect observed in juveniles on the Y maze performance, while several stress effects we found here were absent in juveniles, thus underlining the importance of behavioral testing of Fmr1-KO mice at multiple ages for the study of gene-environment interactions.

## Conclusion

The detrimental behavioral effects of prenatal stress observed here in mutant and WT mice supported the role of unpredictable chronic stress exposure during the last phase of pregnancy as a powerful tool to investigate the contribution of gene-environment interactions in mouse models of mental disorders, especially those of neurodevelopmental nature. As postulated by previous theories (Grossman et al., 2003), aversive influences, arising from genetic and/or environmental sources, could operate on the process of brain development and therefore on an individual’s developmental progression in a disruptive manner, i.e., leading the process away from the normative developmental pathway. Our findings (in particular those concerning Y-maze data from old mice) indeed suggest that prenatal stress exposure may be the necessary additional hit to the emergence of age-specific behavioral deficits. Our findings also underline the relevance of aging as a life phase of high interest to study the impact of genetic and environmental insults, as well as their interactions, in mouse models of neurodevelopmental syndromes. Overall, the behavioral effects of stress as well as those of the Fmr1 mutation appeared slightly more pronounced at aging than at adulthood, thus highlighting the importance of including the aging phase in future studies on the Fmr1-KO mouse model.

## Data availability statement

The raw data supporting the conclusions of this article will be made available by the authors, without undue reservation.

## Ethics statement

The animal study was reviewed and approved by “Comité d’Ethique pour l’experimentation animale de Bordeaux,” CE 50 and the French Ministry (“Ministére de l’Enseignement supérieur, de la Recherche et de l’Innovation”).

## Author contributions

VP analyzed all the behavioral data, prepared all figures, and wrote the results. ES performed part of the experimental work. MP scored the social interaction data. MM contributed to manuscript writing. VL designed and performed the experimental work and supervised data analysis. SP designed the experiments, supervised data analysis, and wrote the manuscript. All authors reviewed and approved the manuscript.
